# Female Zebra Finches Smell Their Eggs

**DOI:** 10.1371/journal.pone.0155513

**Published:** 2016-05-18

**Authors:** Sarah Golüke, Sebastian Dörrenberg, E. Tobias Krause, Barbara A. Caspers

**Affiliations:** 1 Research Group Olfactory Communication, Department of Animal Behaviour, Bielefeld University, Bielefeld, Germany; 2 Friedrich-Loeffler Institut, Institute of Animal Welfare and Animal Husbandry, Celle, Germany; Hungarian Academy of Sciences, HUNGARY

## Abstract

Parental investment in unrelated offspring seems maladaptive from an evolutionary perspective, due to the costs of energy and resources that cannot be invested in related offspring at the same time. Therefore selection should favour mechanisms to discriminate between own and foreign offspring. In birds, much emphasis has been placed on understanding the visual mechanisms underlying egg recognition. However, olfactory egg recognition has almost been completely ignored. Here, we investigated whether female zebra finches (*Taeniopygia guttata*) are able to discriminate between their own and a conspecific egg based on olfactory cues alone. Zebra finches are colonial—breeding songbirds. Eggs are monomorphic, i.e. without any spotting pattern, and intraspecific brood parasitism frequently occurs. In a binary choice experiment, female zebra finches were given the choice between the scent of their own and a conspecific egg. After the onset of incubation, females chose randomly and showed no sign of discrimination. However, shortly before hatching, females preferred significantly the odour of their own egg. The finding that females are capable to smell their own egg may inspire more research on the potential of olfaction involved in egg recognition, especially in cases where visual cues might be limited.

## Introduction

Raising offspring until independence is one of the major challenges in species with parental care. Because parental care is costly in terms of time and energy, it should be provided exclusively to descendent young [1]. Accordingly, selection should have favoured mechanisms in adults to discriminate between own and foreign offspring [2]. Interspecific brood parasitism is probably the most prominent and obvious example in which individuals of some species seem to have failed to exhibit a recognition mechanism. However, birds are faced not only with interspecific brood parasites, such as cuckoos and cowbirds [3–5], but also with conspecific brood parasites [6], which Davies describes as “cheating on your own kind” [4].

Although much emphasis has been placed on understanding the mechanisms by which hosts recognise interspecific brood parasites [7–9], less effort has been directed towards understanding if and how parents recognise conspecific brood parasitism (CBP) [10].

However, at least 234 species show conspecific brood parasitism, and this number is likely to be an underestimation [11]. Colonial—breeding species are most vulnerable to CBP [11] because a high density of nests offers a greater opportunity for CBP and facilitates the correct timing of egg laying [12].

The cost of raising a conspecific chick within the own brood is likely to be smaller compared to the potential fatal consequences of being the host of evicting interspecific brood parasites [4]. Therefore, the selection pressure of recognising CBP is assumed to be lower [13,14]. In addition, intraspecific variation in the appearance of eggs is generally supposed to be lower than interspecific variation, and discriminating own from conspecific eggs should thus be even more challenging [15]. Such potential intraspecific variation, however, referred mostly to egg colouration or spotting patterns.

One potential mechanism for egg recognition that has so far received little attention is the use of olfactory cues. The first evidence of olfactory egg recognition in birds came from a study of magpies (*Pica pica*) [16]. Magpies discriminate against eggs with an odd smell, which might be a mechanism for detecting and rejecting parasitic eggs [16]. Furthermore, it has recently been shown that volatiles emitted from quail eggs (*Cotrunix japonica*) differ between fertilised and unfertilised eggs and even between eggs containing males or females [17].

These findings raise the question whether olfaction may play a role in egg discrimination and recognition, especially in circumstances where visual differences are negligible, as expected in CBP. Following this idea, we tested whether zebra finch females are able to discriminate their own from conspecific eggs by olfactory cues. The zebra finch (*Taeniopygia guttata*) is suitable for a study for discrimination against conspecific eggs for two reasons: they live in dense colonies [18], where the proportion of CBP is expected to be high [11], and occurs quite regularly. The reported conspecific parasitism rates in the wild vary from 17.5% of clutches and 5.4% of offspring [19] up to 36% of clutches and 11% of offspring [20]. With 21% of clutches and 5.4% of offspring [21], the CBP rate of lab populations is in this range. In addition, zebra finches lay monomorphic white eggs in dark domed nests [18,22], which could make it more difficult to use visual cues for egg discrimination [23].

To test whether odours may be involved in egg recognition, we presented zebra finch females, in a simultaneous binary choice situation, the odour of one of their own eggs and a conspecific egg. Zebra finches are known to use olfactory cues for both nest [24–26] and kin recognition [27,28]. If odours are also involved in egg recognition, we expected females to spend more time in the vicinity of the odour of their own egg compared with the conspecifics’ egg odour.

## Material and Methods

### Breeding conditions

Forty-eight male and 48 female zebra finches of the domesticated stock of the University Bielefeld [29] were randomly assigned as breeding pairs, although we avoided any pairings between siblings, parent and offspring, and cousins. The pairs were housed in two compartment cages (80 x 30 x 40 cm) with access to food and water *ad libitum*. Each cage had a nest box (15 x 15 x 15 cm) attached, and coconut fibres were provided as nesting material. The nests were checked daily to assess the start of nest building, the start of egg laying, the completion of the clutch (i.e., when there was no additional egg for two days) and the total number of eggs. Each new egg was weighted (using Kern balances EMB 600–2) and marked at the day of laying. All eggs are treated in exactly the same way. Thirty of the 48 pairs built nests, laid eggs and were included in the analysis. At the end of the experiments, all breeding pairs and their offspring remained in our stock.

### Odour preference test

**Fig 1:**
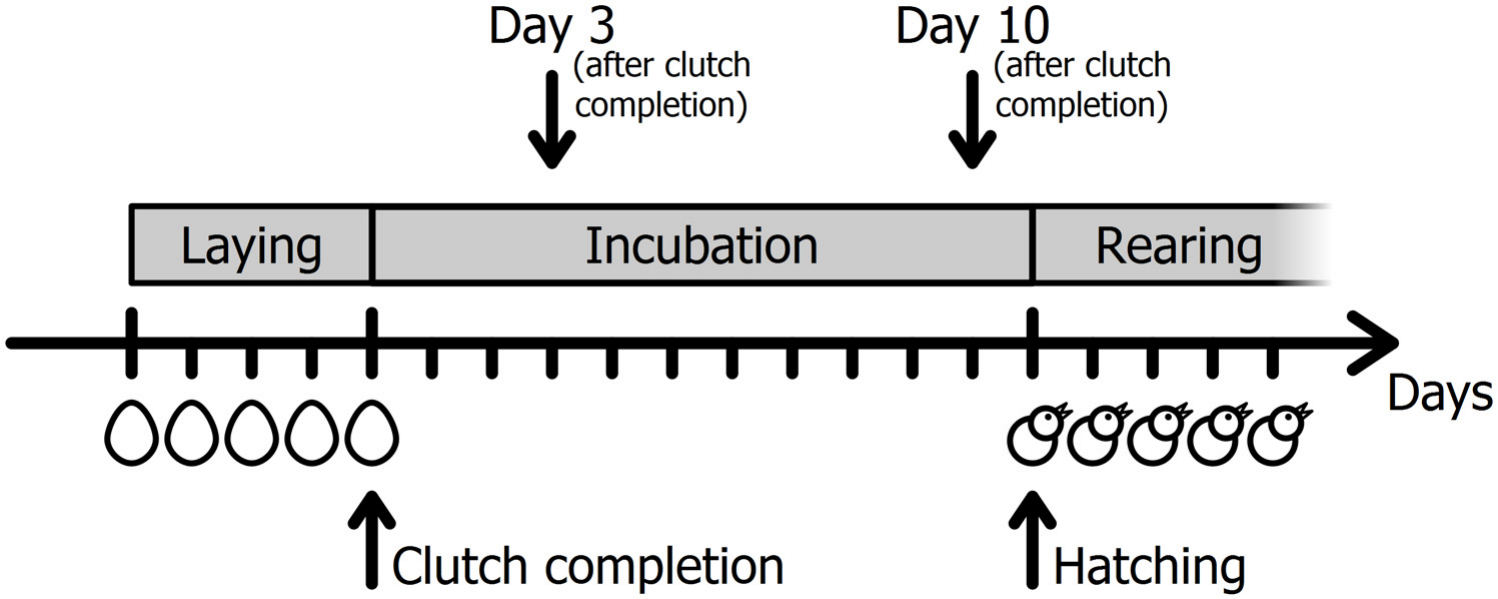
Time scale of the experiment. The time period of egg laying (4–5 days in this dataset) is followed by the time of incubation. Zebra finch chicks hatch after an average of 14 days. The odour preference test was performed on day 3 and 10 after clutch completion.

The aim of this study was to test whether females are able to discriminate between their own and a foreign egg. Therefore, we performed two odour stimulus preference tests. The preference tests were performed on day 3 (± 1 day) and day 10 (± 1 day) after clutch completion (Fig 1). These time points are chosen to guarantee that the females started incubation and not stopped egg laying because of handling, and to ensure, that the eggs did not hatch prior to testing. Zebra finch females usually lay one egg per day and start incubating after clutch completion. Incubation period ranges from 11 to 15 days, with a median of 14 days [18]. Before testing, egg dyads were formed of eggs within an age of 1 day of each other. These dyads were maintained throughout both experimental trials (days 3 and 10), with the exception of two dyads, where we had to exchange one pair because they destroyed their clutch. Whenever possible, the third egg of each clutch was used in the experiment to create a similar age structure for all of the tested eggs. Each egg dyad was used twice as a stimulus pair, i.e., the mothers of both eggs were tested with the same set.

For the duration of the preference test, focal females and stimulus eggs were removed from the home nest box. After and between tests, eggs were placed back into their respective nests. In zebra finches, the male often incubates in the absence of the female. Therefore, eggs that were taken as stimuli were replaced by plastic dummy eggs, which were exchanged again after testing. Because the birds might react to human odour on the egg surface [16], the eggs were only touched with gloves. To exclude the possibility of transferring olfactory cues from one egg to another, gloves were changed every time before handling a new egg.

**Fig 2:**
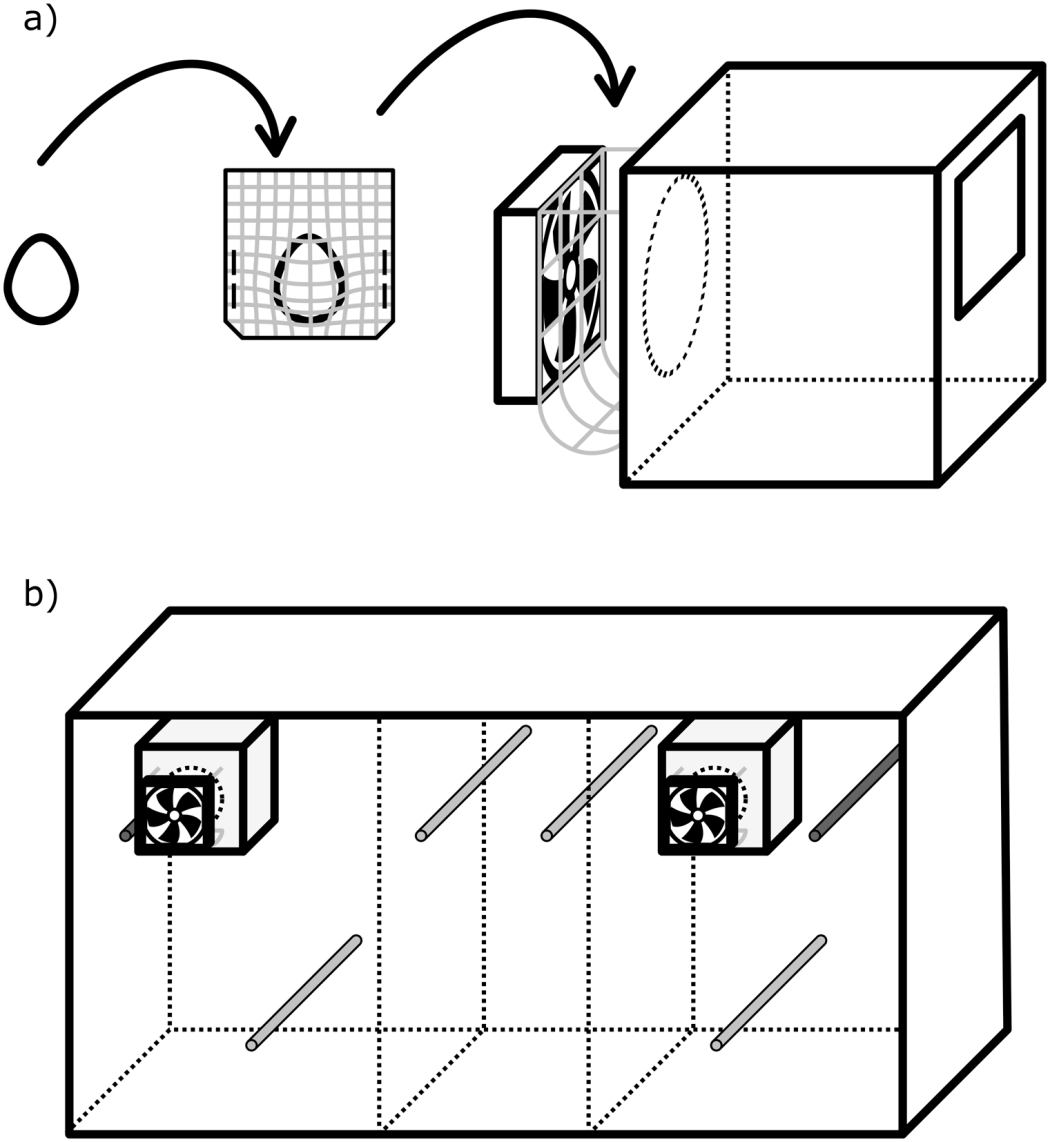
Schematic overview of the odour preference test procedure. A) Scheme of the experimental nest box. Each egg that was tested was taken from the nest box and transferred into a bag made of synthetic gauze. These bags were placed into a wire mesh basket located between the hole at the back of the experimental nest box and the fan. Prior and during the experiment the fan produced an airstream that transferred the odour of the egg though the nest box. B) The odour preference test setup. A three compartment cage was used, equipped with two experimental nest boxes, as shown in A. Prior to the experiment, during the habituation period of the test female, two opaque slides prevented the female from entering the side compartments (dashed lines). Each test consisted of two parts. After the first part, we switched the egg-containing gauze bags between the two experimental nest boxes to control for potential side preferences. We counted the time a bird spent in each of the two preference zones, i.e. the specific nest box, and the perch in front of it (dark grey).

The odour preference test took place in a three-compartment cage (115 x 40 x 30 cm), with one experimental nest box (15 x 15 x 15 cm) attached to each side (Fig 2). The nest boxes were filled with fresh, unused coconut fibres, imitating a nest. In the back wall of each nest box was a round hole (diameter 7.5 cm), covered by wire mesh, with a wire mesh basket behind (see Fig 2A or [25]). The stimulus eggs were individually placed in a single-used bag made of synthetic gauze. Each bag containing one egg was transferred into the wire mesh basket, behind the hole of one of the nest boxes. A fan (Sunon 40 x 40 x 10 cm, 12 volt reduced to 9 volt) was installed behind the wire mesh basket, which created a continuous airflow that transported the egg odour through the hole into the nest box and the test cage. The nest material in the nest box covered the hole in the wall and prevented the females from having visual contact with the eggs. Furthermore, a possible impact of acoustic cues seems very unlikely as zebra finch nestlings do not vocalise until the third day after hatching [18].

The testing procedure was the same as described by Caspers & Krause (2011) [25]. Briefly, before introducing the females to the test cage (Fig 2B), the two fans were turned on for 20 min to allow the accumulation of egg odours in each of the side compartments. To prevent the two odours from mixing, each of the side compartments was separated from the middle compartment by an opaque slide. During the time of odour accumulation, one female was introduced into the middle compartment, which was separated both visually and olfactorily from the other two compartments by opaque slides. The females were allowed to acclimatise for at least 5 min before the opaque slides were removed and the test started. Each female was tested for 5 min, and her position and whether she had moved were recorded every 3 s. Each nest box and the adjacent perch were defined as preference zones. After the test, the female was removed, and the test was repeated with the second female.

To control for side effects, the odour preference test was repeated after switching the odour samples. Therefore, the opaque slides were put into the cage to separate the three compartments, the female (this time we started with the second female first) was placed in the central compartment, and the fans were turned on for another 20 min to allow the accumulation of egg odours in the side compartments. After 20 min of odour accumulation, the opaque slides were removed, and the second half of the test started. Again, each female was tested for 5 min, and her position and whether she had moved were recorded every 3 s. After the second half of the test, the females were released into their home cages, and the eggs were laid back in their original nest. None of the pairs rejected the stimulus egg after replacing it in the home nest.

The observer followed the experiments using two video cameras and a quad-monitor (ELV electronics, Leer, Germany), to guarantee that the test female was not disturbed by the experimenter’s presence. A minimum of four other neutral females were placed symmetrically, but without visual contact with the focal female, in the test room to enhance the background noises and to reduce stress for the focal females.

The time that the female spent in each preference zone was calculated for each female and for each trial following the procedure described by Witte & Caspers (2006) [30], i.e., in case the focal female moved during the 3 s intervals, 1.5 s was scored. If she did not move during the interval, 3 s was scored. The scores were summed and combined for both trials.

### Statistical analysis

The time that a female spent in proximity of either the own or the conspecific egg was analysed using a generalised linear model (GLM) with quasi-binomial distribution, comparing whether the time that a female spent in the vicinity of the own egg differed significantly from the time expected under random choice. Independent models were made for days 3 and 10, as the group of tested females differed somewhat between the trials. Egg weight did not affect female choice and was therefore excluded in the subsequent analysis for both days (see results), using backward selection to get the minimal adequate model.

GLMs were fitted in R version 3.1.2 [31] using the lme4 package version 1.1–7 [32]. The results are given as the means ± SD unless noted otherwise.

### Ethical note

Housing and breeding of birds was approved by the Gesundheits-, Veterinär- und Lebensmittelüberwachungsamt der Stadt Bielefeld (#530.421630–1,18.4.2002). Conditions are assumed to be superior to natural conditions because all animals had *ad libitum* food. All animals were checked daily to verify that the individuals were healthy.

## Results

In total, 31 females were tested for their ability to discriminate between their own and a conspecific egg in the odour preference test. The majority (24 of 31) of the females were tested on both time points. Four females were tested on day three after clutch completion only, and four females were tested on day ten after clutch completion only.

The mean clutch size of the thirty breeding pairs was 4.87 ± 1.26 eggs, in the range of two to eight eggs. The time span between egg laying and hatching per egg was, on average, 14.05 ± 0.83 days. The overall egg weight was 0.95 ± 0.17 g. The females did not discriminate between eggs based on egg weight, as the mean egg mass was 0.93 ± 0.20 g for the preferred and 0.95 ± 0.10 g for the non-preferred eggs on day three; it was 0.96 ± 0.17 g for the preferred eggs on day 10 and 0.95 ± 0.14 g for the non-preferred eggs. The female decision was influenced by the egg weight on neither day three (GLM: t = 0.008, df = 23, p = 0.993) nor day 10 of incubation (GLM: t = 0.19, df = 23, p = 0.85), and the egg weight was therefore excluded from the analysis.

### Early incubation period: Day 3

During the test conducted three days after clutch completion, two of the 27 females did not move into one of the two preference zones and were excluded from the subsequent analysis, leading to a sample size of 25 females.

**Fig 3:**
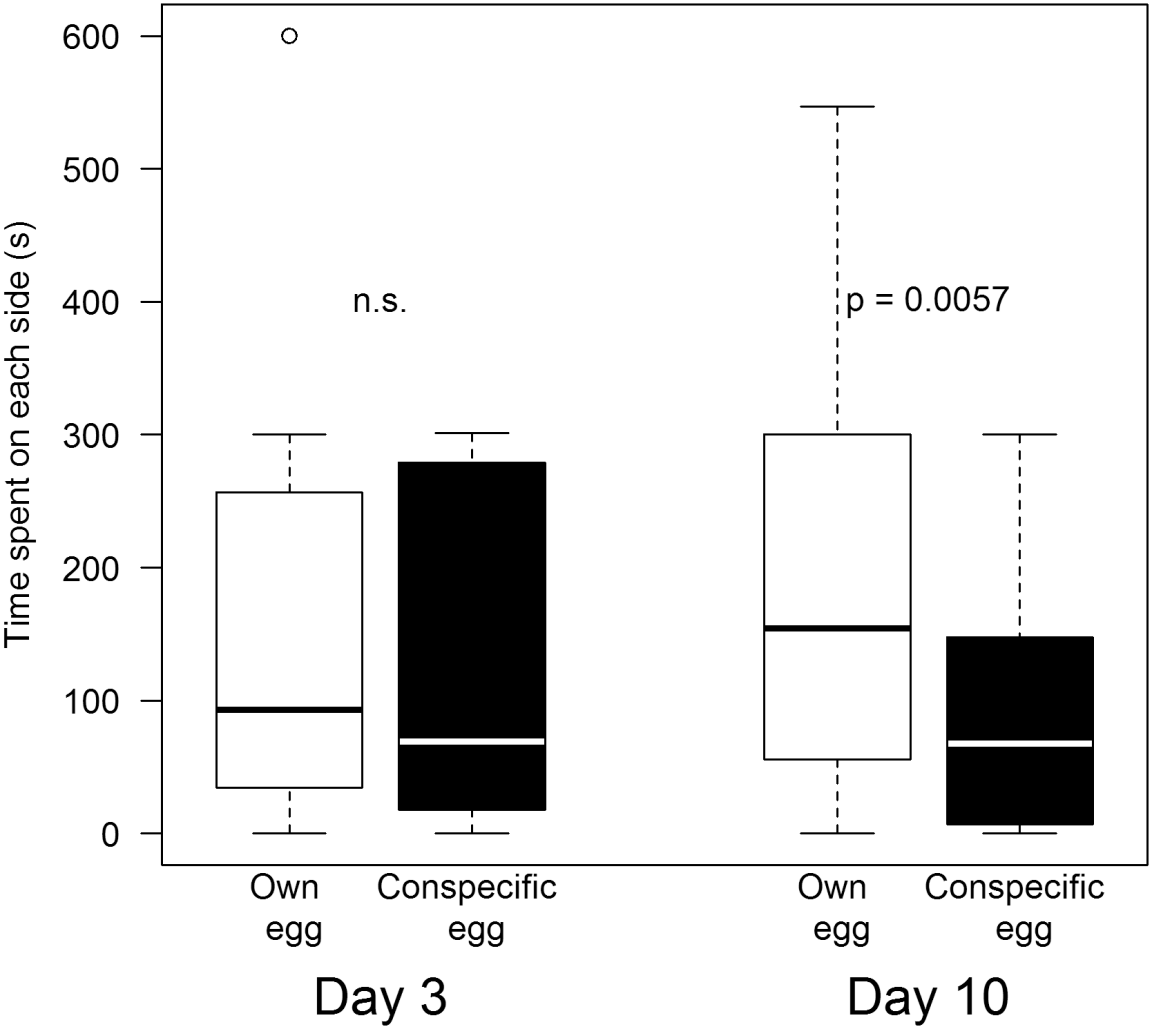
Results of the odour preference tests. Time in seconds that females spent in the preference zone for their own (white) and the conspecific (black) egg odour during early incubation (day 3 after clutch completion, left) and late incubation (day 10, right).

The females did not differ in the amount of time that they spent in the two preference zones (own egg: Median 93 s, conspecific egg: Median: 69 s; GLM: *t*_*1*,*24*_ = 0.25, *p* = 0.802, Fig 3), thus showing no sign of discrimination.

### Late incubation period: Day 10

During the test conducted on day ten, three of the 28 females did not move to either side and were excluded from the analysis. In total, 25 females were analysed for their choice during late incubation.

Here, females spent significantly more time in proximity of their own egg odour (own egg: Median 154.5 s, conspecific egg: Median 67,5 s; GLM: *t*_*1*,*23*_ = 3.034, *p* = 0.0057; Fig 3).

## Discussion

Although much emphasis has been placed on understanding the mechanisms underlying egg recognition in birds, the use of olfactory cues for egg recognition has only recently drawn attention. Our results demonstrate that zebra finch females recognise their own egg shortly before the chicks hatch, as they spent significantly more time in the vicinity of the odour of their own egg, compared to the conspecific eggs. Although our experiment did not allow to answer the question whether olfactory egg recognition is a mechanism to recognise parasitic eggs, the finding that females are capable to recognise their eggs at the end of the incubation period underlines the olfactory abilities of birds and hopefully inspires more researchers to investigate the potential of olfaction in egg recognition.

To facilitate olfactory egg recognition at the end of the incubation period, the chemical profiles of eggs originating from different nests and/or females have to differ. The differences in the odour profiles between eggs can result either from olfactory cues emitted from the developing embryo inside the egg or from substances that are transferred during incubation on the egg surface; however, these mechanisms may not be mutually exclusive.

During embryonic development, eggs emit volatile compounds that differ according to the sex of the embryo [17], i.e., eggs containing male embryos emitted significantly different volatiles than did eggs containing female embryos. It may also be possible that embryos emit heritable-influenced (individual or family specific) volatiles, which enable females to distinguish between eggs.

Although female starlings (*Sturnus unicolor*) do not show a sign of discrimination between own and conspecific offspring based on olfactory cues [33], adult penguins (*Spheniscus humboldti*) are known to distinguish between conspecifics according to familiarity or relatedness [34]. Until now, there is nothing known about olfactory cues of familiarity deriving from eggs.

Furthermore, mother- or family-specific differences in egg odours can be the result of egg preening, i.e., the continuous transfer of odorous substances onto the eggshell. For example, female wood hoopoes actively preen their eggs with uropygial gland secretions [35–37]. Uropygial gland secretions are well known to emit volatile compounds that are important for chemical communication [38–41] and are consistent over years [42]. Likewise, zebra finches, as most if not all other bird species, may also transfer preen gland secretions to their eggs during incubation, either directly as hoopoes do or indirectly via body contact with preened plumage during incubation. In this case, recognition would be based on their own smell and would consequently not allow the detection of conspecific brood parasitism as the female would mask a dumped egg with their own preen gland secretion.

In addition, eggs are known to absorb volatiles from the environment [43] and may also absorb nest odours while lying in the nest. Some species are known to discriminate their own or their mates odour from conspecific scents [44–46], and zebra finches are able to discriminate between kin and non kin [27] and between own and foreign nests [25] by olfactory cues. The discrimination of eggs based on the absorption of nest odours is therefore a possible mechanism.

Finally, eggs may have a unique signature that might be transferred onto the eggshell during egg laying, similar to the assumption of a unique visual signature [47,48]. However, we assume that this is rather unlikely. If unique signatures were present from the moment of egg laying, we would expect females to discriminate between eggs from the time of laying onward, which they did not do.

In contrast to the late incubation period, shortly after clutch completion, the zebra finch females do not show any discrimination between their own egg and a conspecific egg. A lack of discrimination may be due to the absence of cues that are important in egg recognition. The cues needed for recognition may simply not be developed shortly after the onset of incubation: Independent of whether females may use cues from the inside or the outside of the egg, both need time to develop. The developing embryo is very small at three days after starting incubation, and in case females preen their eggs, the amount of preen gland secretion on the egg should be lower compared to that found at day 10, thus making egg recognition more difficult at that early stage.

Although we can only speculate about the origin of chemical differences, the ability to discriminate between own and conspecific eggs raises the question whether olfactory egg recognition is used to recognise eggs from brood parasites. The ability to distinguish between own and foreign eggs at day 10 might be early enough, as the chicks are not yet hatched. Whereas incubation is costly in terms of time and energy demands [49], the cost of incubating an additional egg should be lower than the risk of rejecting a wrong egg. Therefore, the motivation of the females at the two stages of incubation might differ and thus lead to differences in odour discrimination. The discrimination behaviour of female zebra finches might depend on the stimulus as well as on the motivation of the female [24].

Apart from the fact that the molecular analysis revealed relatively high levels of CBP in zebra finches [19–21], nothing is known about whether or how zebra finches reject specific eggs. Brood abandoning, which can be seen as a consequence of interspecific brood parasitism [6,50] may be one possibility, but it seems rather unlikely at that late stage of incubation [51]. Egg rejection in the form of burying [15,52], destroying or ejecting [50] a specific egg from the nest would be more efficient, but currently for zebra finches, no data is available to support one of these ideas. Hatching failure occurs in zebra finches [53], which may be a result of fertilisation failure or a rejection from breeding and leaves space for speculation.

## Conclusion

Zebra finch females are able to discriminate between their own egg and a conspecific egg based on olfactory cues alone, indicating that eggs exhibit different chemical fingerprints and underlining the olfactory sensitivity of birds in specific situations. This study is a first step and shows the potential of odours involved in egg recognition, a thus far neglected egg recognition mechanism. Further studies are necessary to investigate whether olfactory egg recognition is involved in inter- and intraspecific brood parasitism.

## References

[1] Royle NJ, Smiseth PT, Kölliker M. The evolution of parental care. Oxford: Oxford University Press; 2012.

[2] Dawkins R, Krebs JR. Arms races between and within species. Proc R Soc Lond B. 1979;205: 489–511. 42057 10.1098/rspb.1979.0081

[3] Kilner RM, Langmore NE. Cuckoos versus hosts in insects and birds: Adaptations, counter-adaptations and outcomes. Biol Rev. 2011;86: 836–852. 10.1111/j.1469-185X.2010.00173.x 21223481

[4] Davies NB. Cuckoos, Cowbirds and Other Cheats. London: T. & A.D. Poyser; 2000 10.1006/anbe.2000.1568

[5] Davies NB, Brooke MDL. Cuckoos versus reed warblers: Adaptations and counteradaptations. Anim Behav. 1988;36: 262–284. 10.1016/S0003-3472(88)80269-0

[6] Petrie M, Møller AP. Laying eggs in others’ nests: Intraspecific brood parasitism in birds. Trends Ecol. Evol. 1991;6: 315–320. 10.1016/0169-5347(91)90038-Y 21232496

[7] Spottiswoode CN, Stevens M. Host-parasite arms races and rapid changes in bird egg appearance. Am Nat. 2012;179: 633–648. 10.1086/665031 22504545

[8] Davies NB, Brooke MDL. An experimental study of co-evolution between the cuckoo, *Cuculus cnorus*, and its hosts. II. Host egg markings, chick discrimination and general discussion. J Anim Ecol. 1989;58: 207–224.

[9] Colombelli-Négrel D, Hauber ME, Robertson J, Sulloway FJ, Hoi H, Griggio M, et al Embryonic learning of vocal passwords in superb fairy-wrens reveals intruder cuckoo nestlings. Curr Biol. 2012;22: 2155–2160. 10.1016/j.cub.2012.09.025 23142041

[10] Lyon BE, Eadie JM. Conspecific brood parasitism in birds: A life-history perspective. Annu Rev Ecol Evol Syst. 2008;39: 343–363. 10.1146/annurev.ecolsys.39.110707.173354

[11] Yom-Tov Y. An updated list and some comments on the occurrence of intraspecific nest parasitism in birds. Ibis. 2001;143: 133 10.1111/j.1474-919X.2001.tb04177.x

[12] Mariette MM, Griffith SC. Conspecific attraction and nest site selection in a nomadic species, the zebra finch. Oikos. 2012;121: 823–834. 10.1111/j.1600-0706.2011.20014.x

[13] Soler M, Ruiz-Castellano C, del C Fernández-Pinos M, Rösler A, Ontanilla J, Pérez-Contreras T. House sparrows selectively eject parasitic conspecific eggs and incur very low rejection costs. Behav Ecol Sociobiol. 2011;65: 1997–2005. 10.1007/s00265-011-1209-z

[14] Yang C, Hu Y, Ma M, Liang W, Møller AP. Absence of egg rejection in an Asian population of house sparrow (*Passer domesticus*), a conspecific brood parasite in Europe. Behav Ecol Sociobiol. 2015;69: 723–727. 10.1007/s00265-015-1886-0

[15] Lyon B. Mechanism of egg recognition in defenses against conspecific brood parasitism: American coots (*Fulica americana*) know their own eggs. Behav Ecol Sociobiol. 2007;61: 455–463. 10.1007/s00265-006-0273-2

[16] Soler JJ, Pérez-Contreras T, De Neve L, Macías-Sánchez E, Møller AP, Soler M. Recognizing odd smells and ejection of brood parasitic eggs. An experimental test in magpies of a novel defensive trait against brood parasitism. J Evol Biol. 2014;27: 1265–1270. 10.1111/jeb.12377 24725170

[17] Webster B, Hayes W, Pike TW. Avian Egg Odour Encodes Information on Embryo Sex, Fertility and Development. PloS ONE. 2015;10: 1–10. 10.1371/journal.pone.0116345 PMC4309571 25629413

[18] Zann RA. The zebra finch: A synthesis of field and laboratory studies. Oxford: Oxford University Press; 1996.

[19] Griffith SC, Holleley CE, Mariette MM, Pryke SR, Svedin N. Low level of extrapair parentage in wild zebra finches. Anim Behav. 2010;79: 261–264. 10.1016/j.anbehav.2009.11.031

[20] Birkhead TR, Burke T, Zann R, Hunter F. M, Krupa AP. Extra-pair paternity and intraspecific brood parasitism in wild zebra finches Taeniopygia gutta, revealed by DNA fingerprinting. Behav Ecol Sociobiol. 1990;27: 315–324.

[21] Schielzeth H, Bolund E. Patterns of conspecific brood parasitism in zebra finches. Anim Behav. 2010;79: 1329–1337. 10.1016/j.anbehav.2010.03.006

[22] Shaw RC, Feeney WE, Hauber ME. Nest destruction elicits indiscriminate con- versus heterospecific brood parasitism in a captive bird. Ecol Evol. 2014;4: 4500–4504. 10.1002/ece3.1243 25512846 PMC4264899

[23] Wesołowski T, Maziarz M. Dark tree cavities—a challenge for hole nesting birds? J Avian Biol. 2012;43: 454–460. 10.1111/j.1600-048X.2012.05704.x

[24] Krause ET, Caspers BA. Are olfactory cues involved in nest recognition in two social species of estrildid finches? PLoS ONE. 2012;7: 1–7. 10.1371/journal.pone.0036615 PMC3344906 22574196

[25] Caspers BA, Krause ET. Odour-based natal nest recognition in the zebra finch (*Taeniopygia guttata*), a colony-breeding songbird. Biol Lett. 2011;7: 184–186. 10.1098/rsbl.2010.0775 20880859 PMC3061170

[26] Caspers BA, Hoffman JI, Kohlmeier P, Krüger O, Krause ET. Olfactory imprinting as a mechanism for nest odour recognition in zebra finches. Anim Behav. 2013;86: 85–90. 10.1016/j.anbehav.2013.04.015

[27] Krause ET, Krüger O, Kohlmeier P, Caspers BA. Olfactory kin recognition in a songbird. Biol Lett. 2012;8: 327–329. 10.1098/rsbl.2011.1093 22219391 PMC3367747

[28] Caspers BA, Gagliardo A, Krause ET. Impact of kin odour on reproduction in zebra finches. Behav Ecol Sociobiol. 2015;69: 1827–1833. 10.1007/s00265-015-1995-9

[29] Hoffman JI, Krause ET, Lehmann K, Krüger O. MC1R genotype and plumage colouration in the zebra finch (*Taeniopygia guttata*): population structure generates artefactual associations. PLoS ONE. 2014;9: 1–9. 10.1371/journal.pone.0086519 PMC3906038 24489736

[30] Witte K, Caspers B. Sexual imprinting on a novel blue ornament in zebra finches. Behaviour. 2006;143: 969–991. 10.1163/156853906778623626

[31] R Core Team. R: A language and environment for statistical computing. Vienna: R Foundation for Statistical Computing; 2014.

[32] Bates D, Maechler M, Bolker B, Walker S. Package “lme4”: Linear mixed-effects models using Eigen and S4_. R package version 1.1–8. 2014;

[33] Amo L, Toma G, Parejo D, Avilés JM. Are female starlings able to recognize the scent of their offspring? PLoS ONE. 2014;9: 1–6. 10.1371/journal.pone.0109505 PMC4192304 25299305

[34] Coffin HR, Watters J V., Mateo JM. Odor-based recognition of familiar and related conspecifics: A first test conducted on captive Humboldt penguins (*Spheniscus humboldti*). PLoS ONE. 2011;6: 10–13. 10.1371/journal.pone.0025002 PMC3177858 21957471

[35] Martín-Vivaldi M, Soler JJ, Peralta-Sánchez JM, Arco L, Martín-Platero AM, Martínez-Bueno M, et al Special structures of hoopoe eggshells enhance the adhesion of symbiont-carrying uropygial secretion that increase hatching success. J Anim Ecol. 2014; 1289–1301. 10.1111/1365-2656.12243 24786478

[36] Martín-Vivaldi M, Ruiz-Rodríguez M, Soler JJ, Peralta-Sánchez JM, Méndez M, Valdivia E, et al Seasonal, sexual and developmental differences in hoopoe Upupa epops preen gland morphology and secretions: Evidence for a role of bacteria. J Avian Biol. 2009;40: 191–205. 10.1111/j.1600-048X.2009.04393.x

[37] Soler JJ, Martín-Vivaldi M, Peralta-Sánchez JM, Arco L, Juárez-García-Pelayo N. Hoopoes color their eggs with antimicrobial uropygial secretions. Naturwissenschaften. 2014;101: 697–705. 10.1007/s00114-014-1201-3 25011415

[38] Campagna S, Mardon J, Celerier A, Bonadonna F. Potential semiochemical molecules from birds: A practical and comprehensive compilation of the last 20 years studies. Chem Senses. 2012;37: 3–25. 10.1093/chemse/bjr067 21798850

[39] Caro SP, Balthazart J, Bonadonna F. The perfume of reproduction in birds: Chemosignaling in avian social life. Horm Behav. 2014;68: 25–42. 10.1016/j.yhbeh.2014.06.001 24928570 PMC4263688

[40] Hagelin JC, Jones IL. Bird odors and other chemical substancees: A defense mechanism or overlooked mode of intraspecific communication? Auk. 2007;124: 741–761.

[41] Whittaker DJ, Gerlach NM, Soini HA, Novotny MV, Ketterson ED. Bird odour predicts reproductive success. Anim Behav. 2013;86: 697–703. 10.1016/j.anbehav.2013.07.025

[42] Mardon J, Saunders SM, Anderson MJ, Couchoux C, Bonadonna F. Species, gender, and identity: Cracking petrels’ sociochemical code. Chem Senses. 2010;35: 309–321. 10.1093/chemse/bjq021 20190009

[43] Maga JA. Egg and egg product flavor. J Agric Food Chem. 1982;30: 9–14. 10.1021/jf00109a002

[44] Bonadonna F, Nevitt GA. Partner-specific odor recognition in an Antarctic seabird. Science. 2004;306: 2004 10.1126/science.1103001 15514149

[45] De León A, Mínguez E, Belliure B. Self-odour recognition in European storm-petrel chicks. Behaviour. 2003;140: 925–933.

[46] Whittaker DJ, Richmond KM, Miller AK, Kiley R, Bergeon Burns C, Atwell JW, et al Intraspecific preen oil odor preferences in dark-eyed juncos (*Junco hyemalis*). Behav Ecol. 2011;22: 1256–1263. 10.1093/beheco/arr122

[47] Cherry MI, Bennett ATD, Moskát C. Host intra-clutch variation, cuckoo egg matching and egg rejection by great reed warblers. Naturwissenschaften. 2007;94: 441–447. 10.1007/s00114-007-0216-4 17252240

[48] Lahti DC, Lahti AR. How precise is egg discrimination in weaverbirds? Anim Behav. 2002;63: 1135–1142. 10.1006/anbe.2002.3009

[49] Monaghan P, Nager RG. Why don’t birds lay more eggs? Trends Ecol Evol. 1997;12: 270–274. 21238065 10.1016/s0169-5347(97)01094-x

[50] Moksnes A, Røskaft E, Braa AT. Rejection behavior by common cuckoo hosts towards artificial brood parasite eggs. Auk. 1991;108: 348–354.

[51] Fenske B, Burley N. Responses of zebra finches (*Taeniopygia guttata*) to experimental brood parasitism. Auk. 1995;112: 415–420.

[52] Lyon B. Egg recognition and counting reduce costs of avian conspecific brood parasitism. Nature. 2003;422: 495–499. 10.1038/nature01505 12673243

[53] Ihle M, Kempenaers B, Forstmeier W. Does hatching failure breed infidelity? Behav Ecol. 2012;24: 119–127. 10.1093/beheco/ars142

